# Classifying Internet Addiction Using Machine Learning Approach: A Study Among Adolescents in Bangladesh

**DOI:** 10.1002/puh2.70165

**Published:** 2025-11-14

**Authors:** Akher Ali, Md. Sahadat Hosain, Md Abu Bakkar Siddik, Mahedi Hasan, Md. Ahashan Habib, Mohammad Alamgir Kabir, Mohammad Mizanur Rahman, Peal Ahamed Shanto, Nafiul Hasan, Al Mahmud

**Affiliations:** ^1^ Department of Statistics and Data Science Jahangirnagar University, Savar Dhaka Bangladesh; ^2^ Department of Development Studies University of Chittagong Chittagong Bangladesh; ^3^ School of the Environment Nanjing University Nanjing China; ^4^ Department of Development Studies Daffodil International University, Savar Dhaka Bangladesh; ^5^ The Center for Social Policy and Justice Dhaka Bangladesh; ^6^ College of Media and Communication Texas Tech University Lubbock Texas USA; ^7^ Department of Business Administration Manarat International University Dhaka Bangladesh; ^8^ Department of Economics Texas Tech University Lubbock Texas USA; ^9^ Department of Statistics Shahjalal University of Science and Technology Sylhet Bangladesh; ^10^ School of Dental Sciences Health Campus Universiti Sains Malaysia Kelantan Malaysia

**Keywords:** confusion matrix, cross‐validation, feature selection, internet addiction, machine learning, receiver operating characteristic (ROC)

## Abstract

**Background:**

Internet addiction (IA) among adolescents is growing worldwide. Online temptation is particularly strong for adolescents due to rapid physical and cognitive development. IA may impair their mental, emotional, social, and physical health. Few traditional studies were conducted in Bangladesh. Thus, this study aimed to identify adolescents’ IA risk factors using advanced machine learning (ML).

**Methods:**

A total of 385 individuals were convenience sampled and surveyed using the Patient Health Questionnaire‐9 (PHQ‐9), the UCLA Loneliness Scale (UCLA‐3), and Young's IA Test (IAT‐20) to measure the prevalence of depression, loneliness, and IA. Boruta found IA prevalence classifying factors. We evaluated decision tree (DT), support vector machine (SVM), logistic regression (LR), and random forest (RF) classification models using confusion matrix, receiver operating characteristic (ROC) curves, and *k*‐fold cross‐validation.

**Results:**

Among 385 respondents, one‐third (30.1%) reported IA. Participants’ fathers’ education, favorite activity, loneliness, smoking status, depression, and internet use time were selected as important features classifying IA. The performance was tested on the basis of five different classification techniques overall: the SVM linear kernel model (accuracy = 0.819, specificity = 0.869, sensitivity = 0.687, precision = 0.666, area under the ROC curve [AUC] = 0.890, *k*‐fold accuracy = 0.801) performed better and authentically classified IA.

**Conclusion:**

Raising awareness among adolescents and their parents is crucial because IA is frequent. The ML framework can identify significant prognostic indicators and classify this IA problem more accurately, helping policymakers, stakeholders, and families understand and prevent this crisis by improving policy‐making strategies and counseling services.

## Graphical abstract

The infographic outlines a study on classifying internet addiction (IA) among adolescents in Bangladesh using machine learning. The study involved 385 participants aged 13–19, focusing on key predictors such as depression, loneliness, smoking status, and internet use time. Data were collected using the IAT‐20, PHQ‐9, and UCLA‐3 scales. The Boruta algorithm was used with models like decision tree, logistic regression, and random forest, achieving an accuracy of 81.9%, sensitivity of 68.7%, specificity of 86.3%, and an AUC of 0.89. The findings highlight significant prevalence rates for internet addiction, depression, and high loneliness among participants.

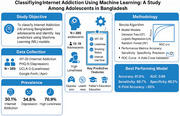

## 1 Introduction

The internet has revolutionized how we live, work, and interact with the world around us. From communication and information access to education and entertainment, the internet has become an indispensable tool that has changed our society [1]. However, its extensiveness has also raised concerns about its potential to foster addictive behaviors [2, 3]. Adolescents are susceptible to internet attraction due to their developmental stage and evolving self‐regulation abilities [4].

In recent years, there has been a significant increase in internet usage in Bangladesh. From 1 million in 2000, internet users have grown to 131 million as of July 2023 [5]. Globally, internet usage among adolescents is growing at a high rate. For example, in a recent study by the Pew Research Center, around 95% of adolescents in the United States use social media, particularly YouTube [6]. Bangladesh is no exception to this. This exponential growth in internet usage has presented a dynamic backdrop against which the interplay between psychological well‐being and online activities unfolds. The escalating prevalence of internet addiction (IA) has emerged as a simultaneous issue, particularly among the youth [7]. As the digital landscape continues to shape social interactions, academic pursuits, and leisure activities, concerns about the impact on mental health, specifically depression and loneliness, have gained prominence [8]. It is crucial to understand the nuanced relationship between the extensive utilization of the internet and its potential impact on the psychological well‐being of individuals, particularly adolescents, amidst the rapid advancement of technology.

The prevalence of major depressive disorder, loneliness, and IA among adolescents varies significantly across different countries. For instance, in the United States, the lifetime prevalence of major depressive disorder among adolescents aged 13–18 is 11.0%, whereas the 12‐month prevalence rate is 7.5% [9]. Regarding loneliness, a comprehensive review of pre‐pandemic studies revealed that the prevalence rates among adolescents aged 12–17 across 76 countries ranged from 9.2% to 14.4%, depending on the geographical location [10]. In the case of IA, the global prevalence rates among adolescents vary widely from 0.4% to 44.7%, primarily due to the absence of a universally accepted definition and standardized measurement tools [11, 12]. Depression and other mental health disorders are very prevalent in Bangladeshi adolescents due to several factors [13, 14, 15]. A cross‐sectional study revealed that depression and anxiety are prevalent among Bangladeshi adolescents, with 26.5% experiencing moderate to severe depression and 18.1% experiencing moderate to severe anxiety. The study identified several associated factors, including insufficient sleep, cigarette smoking, and strained perceived relationships with friends [16]. Machine learning (ML), a revolutionary technology that empowers computers to learn and adapt without explicit programming, has transformed various fields, including healthcare [17, 18]. ML's ability to analyze vast data sets and identify hidden patterns has enabled researchers to classify the complex factors behind depression, loneliness, and IA on a global scale [19]. It also helps researchers pave the way for more effective prevention and intervention strategies. In various countries, scholars are increasingly turning to machine ML algorithms, drawn by their ability to scrutinize vast data sets [20, 21, 22]. These algorithms excel at unveiling patterns and connections that conventional statistical methods may overlook.

This study adopted an innovative ML methodology to classify the factors influencing depression, loneliness, and IA in the context of Bangladesh. This marks a pioneering effort in Bangladeshi academia, as the application of ML techniques for understanding these issues among adolescents is unprecedented in this region. Looking at existing scholarship in Bangladesh, most studies used regression analyses and other relevant statistics and focused on adolescents and college students [23, 24, 25, 26]. But instead of using traditional statistical methods analysis, ML can enhance the new dimensions and findings that will help further understanding. This is one of the first few studies intended to use methods to understand how these mental health things mix up.

The study sought to assess the prevalence of depression, loneliness, and IA among adolescents in Bangladesh, classify the associated factors influencing these psychological states, and determine the relationships between depression, loneliness, and IA by employing the ML approach. Through these objectives, this study addresses the immediate concerns related to adolescents’ mental health and contributes to the broader discourse on the application of ML in understanding psychological phenomena. This study's findings will inform the development of effective prevention and intervention strategies for adolescent mental health in Bangladesh, promoting their overall well‐being and ensuring a brighter future.

## 2 Methodology

### 2.1 Ethical Approval

The study received ethical and logistical approval from Noakhali Science and Technology University's IRB. Reference code: NSTU/SCI/EC/2023/170. Only those who agreed to participate in the research by signing a permission form were included in the analysis.

### 2.2 Sample Size

The study's population comprises adolescents residing in Bangladesh, ranging in age from 13 to 19 years. The sample size required for this study was determined to be 384 persons. This estimating of the desired sample size has been estimated following Cochran's formula: (*n* = *z* ^2^ × *p* × *q*/*d^2^*), utilizing a 95% confidence interval (*d* = 0.05), a critical value of 1.96 for a two‐tailed test (*z* = 1.96), and assuming equal probabilities of success and failure (*p* = 0.5, *q* = 0.5). The research had a total of 385 participants. The post hoc power analysis was performed on the basis of the findings from a sample of 385 individuals. The study's power was determined to be 99% at a 95% confidence level, assuming a medium effect size [27]. The study publication used the STROBE criteria for reporting purposes [28].

### 2.3 Study Design and Data Collection Methods

The 385 participants were recruited between April and June 2023 from several colleges and schools in the Sylhet division, Bangladesh, and employed a cross‐sectional design. To ensure representativeness, participants were selected using a convenience technique. Eligible students were identified from institutional enrollment lists, and each had an equal probability of being selected. This approach was adopted to minimize selection bias and uphold methodological rigor. The sampling strategy has been explicitly clarified in this revised version in response to reviewer feedback.

Initially, a pilot study was conducted with 44 students to assess the feasibility and refine the questionnaire. Following this, the full survey was administered using Google Forms, an efficient online data collection platform. The questionnaire consisted of four sections: demographic information, Young's IA Test (IAT‐20), the Patient Health Questionnaire‐9 (PHQ‐9) for depression, and the UCLA Loneliness Scale (UCLA‐3).

### 2.4 Inclusion and Exclusion Criteria

The inclusion criteria for participants in the study were as follows: (a) enrollment of individuals presently engaged in educational pursuits at the school level; (b) enrollment of individuals currently pursuing education at the college level; (c) inclusion of adolescents below the age of 19; and (d) requirement of Bangladeshi nationality by birth. The exclusion criteria for this study included the following: (a) adolescents who had dual citizenship; (b) international students who were presently enrolled in educational institutions in Sylhet, Bangladesh; (c) adolescents who were currently studying overseas but following the Bangladeshi curriculum.

## 3 Measures

### 3.1 Demographic Measures

Questions regarding socio‐demographics were asked, including father's education (up to school level, up to college level, up to university level, don't know), living with (school/college hostel, with family, with friends/peers in mess, with relatives), average study time (less than 3 h, 4–5 h, more than 5 h), average sleep hour (less than 5 h, 5–6 h, 6–7 h, more than 7 h), device of using internet (mobile, tab, and computer), favorite activities (playing sports, sleeping, using mobile phone, and others), smoking status (yes, no), exercise habit (yes, no), daily average internet use (less than 1 h, 1–2 h, 2–3 h, 3–4 h, 4–5 h, 5–6 h, more than 6 h), relationship with parents (average, good, no), usage of internet as a part of communication (yes, no), usage of internet among parents (yes, no).

### 3.2 Internet Addiction Test Scale

K. Young created the IAT‐20, a standardized internet addiction test scale and one‐dimensional psychometric instrument [29]. The degree of IA was measured by the time individuals spent engaging in internet‐enabled leisure activities across all devices. The IAT consists of 20 multiple‐choice questions scored on a 5‐point Likert scale. Examinees’ IAT scores are calculated by adding their marks for each of the 20 questions. The highest score you can get is 100. Ratings over 50 indicate IA, whereas ratings below 50 indicate no such problem [29, 30].

### 3.3 Patient Depression Questionnaire

Patients with depression were assessed using the PHQ‐9 [31]. The evaluation has nine components, all scored on a Likert scale from 0 to 3. The intensity of depression was classified into four categories, with no symptoms, minimal symptoms, mild symptoms, moderate symptoms, and severe symptoms, on the basis of total scores ranging from 0 to 4 to 9 to 14 to 19 to 27. Depressive symptoms were defined as mild to severe (above 10) in this study's subjects [32]. PHQ‐9 has been widely used in Bangladesh in different settings [33, 34, 35, 36].

### 3.4 UCLA Loneliness Scale

Participants’ subjective loneliness was measured using the revised UCLA loneliness scale (R‐UCLA‐3) [37]. The scale included 20 items, 10 positive and 10 negative, measuring social connection satisfaction and dissatisfaction. Although “lonely” is never mentioned, participants assess many events on a 4‐point Likert scale. Before direction uniformity analysis, 10 favorably phrased questions were reverse rated. After reverse scoring, all questions were averaged to calculate loneliness scores for each participant. Higher scores indicate more loneliness. Scores vary from 20 to 80. The severity cutoffs for loneliness were adopted from Cacioppo and Patrick [38]: <28 = no/low loneliness, 28–43 = moderate loneliness, >43 = strong loneliness [38]. For this research, moderate to high loneliness was considered loneliness.

### 3.5 Statistical Analysis

The goal of this study was to evaluate the risk factors and classify IA among Bangladeshi adolescents by applying various ML models such as evaluated decision tree (DT), support vector machine (SVM), logistic regression (LR), and random forest (RF). After data collection, the data were coded, cleaned, and prepared for final analysis using Microsoft Excel 19. After that, the Excel file was imported into R programming software, where the reliability of the data was tested. The value of Cronbach's alpha was 0.78. Then descriptive and inferential statistics (chi‐square) were initially used. Our methodology entails the appropriate data collection and preprocessing, feature (the risk factors) selection using the Boruta algorithm, splitting the entire data set into training and test data sets—applying ML models in the training data set and assessing these models’ performance on the test data sets—and finally using the model that performed the best to classify IA based on the entire data set. The performances were evaluated using four performance parameters from the confusion matrix: sensitivity, specificity, accuracy, the area under the receiver operating characteristic (ROC) curve (AUC), and the *k*‐fold cross‐validation. All ML models were performed using the scikit‐learn module in Python programming language version 3.7.3. The Boruta algorithm was implemented to select the risk factors using the “Boruta” package library in the R programming language.

### 3.6 Rationale for Applying ML in Public Health Research

ML methods, such as DT, SVM, LR, and RF, are well‐suited for analyzing perceived IA due to their ability to handle complex, nonlinear relationships in behavioral data. These models can effectively identify key predictors from a wide range of psychological, social, and demographic features. DT and RF provide interpretability and feature importance, making them valuable for understanding decision paths. SVM excels in high‐dimensional classification, whereas LR offers a baseline with probabilistic interpretations. Their combined use enhances prediction accuracy and reliability in adolescent behavioral studies.

## 4 Results

### 4.1 Descriptive Characteristic Cross‐Tabulation of the Participants

A total of 385 adolescents participated in the survey; 116 were internet addicted (30.1%). Among the total participants, 137 (35.6%) respondents whose father's education level was up to school were followed by 67 (17.4%) respondents whose father's education level was up to college level. A total of 348 (90.4%) lived with their families, followed by 5 (1.3%) of those who lived in school/college/hostel. Most students, 122 (31.7%), had an average study time of 3–4 h, followed by 72 (18.7%) who studied for more than 5 h. Just over one‐third of the adolescents, 138 (35.8%), had an average sleep time of 6–7 h, followed by 54 (14%) were less than 5 h. Of most of them, 337 (87.5%) were used in mobile devices, followed by 23 (6%) used in tablet devices. Among all respondents, 20 (5.2%) were smokers, 132 (34.3%) played games on the internet, and 210 (54.5%) practiced exercise. The sample included 158 (41%) who used internet time for less than 1 h, followed by 6 (1.6%) who used internet time for 5–6 h. The highest proportion of respondents, 361 (93.8%), had a good relationship with their parents; among a total of 337 (87.5%), they used the internet to connect with close friends, and 249 (64.7%) respondents had parents who used the internet. The highest proportion, 273 (70.9%), had a high loneliness status, followed by 8 (2.1%) had low loneliness status. Among the total, 134 (34.8%) were depressed, and the remaining 251 (65.2%) were not depressed, as shown in Table 1.

**TABLE 1:** Frequency distribution and relationship with internet addiction among adolescents.

Variables	Category	Total 385 *n* (%)	Internet addicted *n* = 116 (30.1%)		
			Yes (%)	*χ* ^2^	*p* value
Father's education	Don't know	96 (24.9)	27 (28.1)	20.13	<0.01***
	Up to school	137 (35.6)	25 (18.2)		
	College	67 (17.4)	30 (44.8)		
	University	85 (22.1)	34 (40)		
Lives with	School/College/Hostel	5 (1.3)	3 (60)	2.56	FET = 0.479
	Family	348 (90.4)	105 (30.2)		
	Friends	15 (3.9)	4 (26.7)		
	Relatives	17 (4.4)	4 (23.5)		
Average study time	<3	93 (24.2)	34 (36.6)	4.09	0.252
	3–4 h	122 (31.7)	30 (24.6)		
	4–5 h	98 (25.5)	32 (32.7)		
	>5 h	72 (18.7)	20 (27.8)		
Sleep hour	<5 h	54 (14)	17 (31.5)	2.33	0.506
	5–6 h	87 (22.6)	27 (31)		
	6–7 h	138 (35.8)	46 (33.3)		
	>7 h	106 (27.5)	26 (24.5)		
Device of using internet	Mobile	337 (87.5)	105 (31.2)	1.38	0.5
	Tab	23 (6)	5 (21.7)		
	Computer	25 (6.5)	6 (24)		
Favorite activity	Gaming	14 (3.6)	9 (64.3)	62.38	<0.01***
	Hanging around	120 (31.2)	27 (22.5)		
	Playing	83 (21.6)	16 (19.3)		
	Sleeping	73 (19)	27 (37)		
	Using mobile phone	36 (9.4)	28 (77.8)		
	Others	59 (15.3)	9 (15.3)		
Smoking status	No	365 (94.8)	102 (27.9)	15.93	<0.01***
	Yes	20 (5.2)	14 (70)		
Internet games	No	253 (65.7)	42 (16.6)	64.16	<0.01***
	Yes	132 (34.3)	74 (56.1)		
Exercise	No	175 (45.5)	58 (33.1)	1.38	0.24
	Yes	210 (54.5)	58 (27.6)		
Internet use time	<1 h	158 (41)	15 (9.5)	109.5	FET = 0.00***
	1–2 h	85 (22.1)	17 (20)		
	2–3 h	45 (11.7)	20 (44.4)		
	3–4 h	45 (11.7)	26 (57.8)		
	4–5 h	22 (5.7)	15 (68.2)		
	5–6 h	6 (1.6)	2 (33.3)		
	>6 h	24 (6.2)	21 (87.5)		
Relationship with parents	Average	24 (6.2)	11 (45.8)	3	0.083
	Good	361 (93.8)	105 (29.1)		
Internet used to connect with close friends	No	48 (12.5)	3 (6.3)	14.85	FET = 0***
	Yes	337 (87.5)	113 (33.5)		
Parents use internet	No	136 (35.3)	33 (24.3)	3.44	0.064
	Yes	249 (64.7)	83 (33.3)		
Loneliness	Low	8 (2.1)	0 (0)	17.72	FET = 0***
	Modern	104 (27)	17 (16.3)		
	High	273 (70.9)	99 (36.3)		
Depression	No	251 (65.2)	37 (14.7)	81.13	<0.01***
	Yes	134 (34.8)	79 (59)		

Table 1 also exhibits that respondent father's education (*χ* ^2^ = 20.13, *p* value <0.01), favorite activity (*χ* ^2^ = 62.38, *p* value <0.01), smoking status (*χ* ^2^ = 15.93, *p* value <0.01), internet games (*χ* ^2^ = 64.16, *p* value <0.01), internet use time (FET = 0.01***), internet used to connect close friend (FET = 0.01***), loneliness (FET = 0.01***), and depression (*χ* ^2^ = 81.13, *p* value <0.01) were significantly associated with IA.

### 4.2 Feature Selection

**FIGURE 1:**
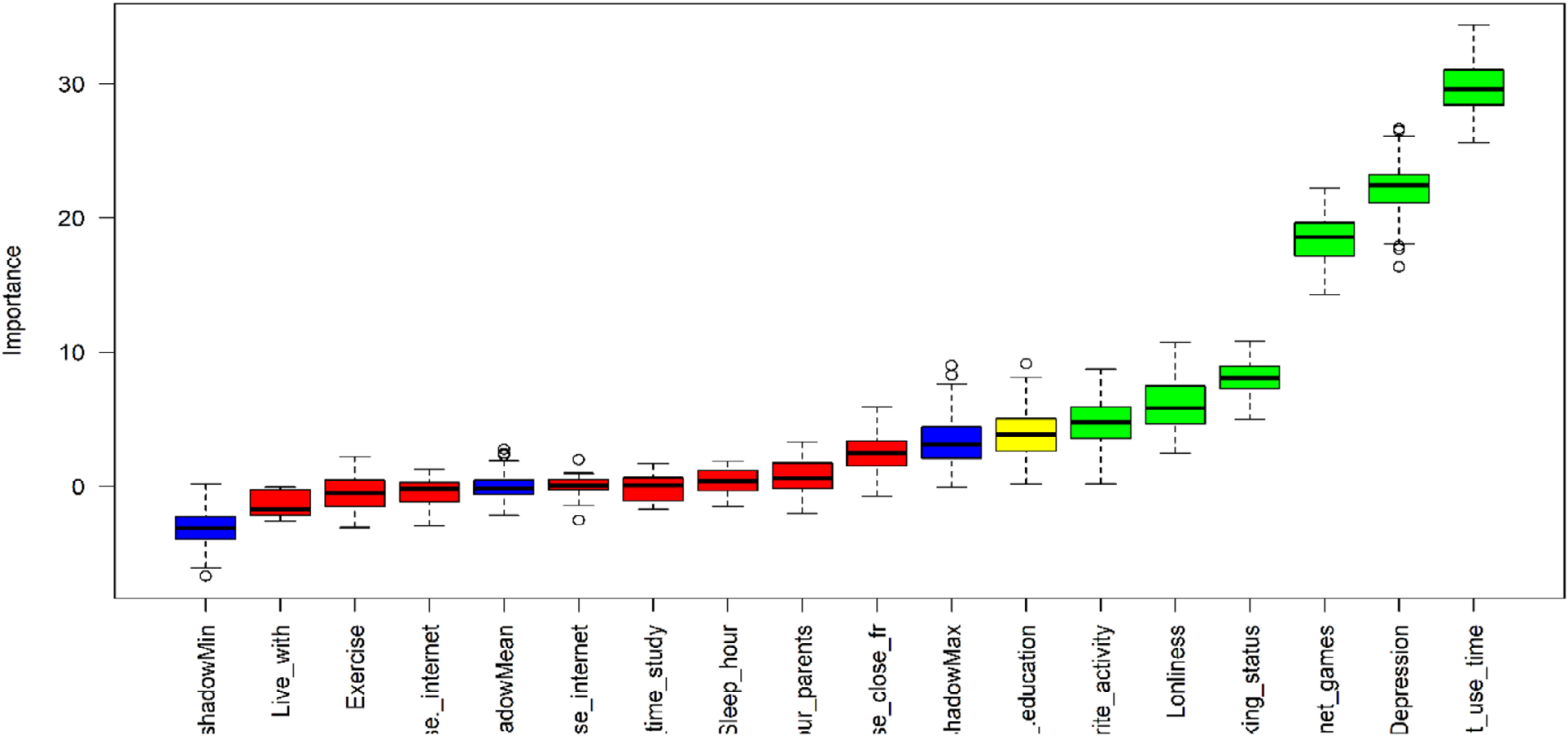
Feature selection using the Boruta algorithm.

Figure 1 reveals that with the air of the Boruta algorithm, six features (favorite activity, loneliness, smoking status, depression, and internet use time) were selected among 15 features as risk factors to classify IA among adolescents. Students’ favorite activity, loneliness, smoking status, depression, and internet use time were confirmed features, and the father's education was a tentative feature for classifying their IA. Hereafter, these seven features were used to evaluate the performance of the ML algorithm.

Table 2 provides a summary of the confirmation table for IA, displaying key metrics including minimum importance, maximum importance, mean importance, median importance, normhist value, and corresponding decision outcomes. From this confirmation table, seven features were confirmed for IA among adolescents, and these seven features were used to evaluate the performance of the ML algorithm.

**TABLE 2:** Confirmation table by using Boruta algorithm.

Feature	meanImp	medianImp	minImp	maxImp	normHits	Decision
Father's education	4.14	4.37	−0.28	9.29	0.65	Confirmed
Live with	−2.02	−2.42	−3.50	1.36	0	Rejected
Average time study	−0.10	0.03	−3.06	3.40	0.01	Rejected
Sleep hour	−0.43	−0.54	−1.88	1.29	0	Rejected
Device use internet	0.14	0.27	−2.10	2.40	0	Rejected
Favorite activity	4.35	4.01	0.87	8.91	0.74	Confirmed
Smoking status	7.88	7.78	5.70	11.73	1	Confirmed
Internet games	18.26	18.24	14.30	23.70	1	Confirmed
Exercise	0.07	0.13	−2.31	3.02	0.01	Rejected
Internet use time	29.52	29.52	25.77	35.55	1	Confirmed
Relationship with parents	0.75	0.87	−1.95	3.11	0.05	Rejected
Internet use by close friend	2.40	2.34	−0.76	6.59	0.32	Rejected
Parents use internet	0.13	0.36	−2.31	3.46	0.01	Rejected
Loneliness	5.99	5.99	2.06	9.62	0.89	Confirmed
Depression	21.96	22.04	17.82	27.56	1	Confirmed

### 4.3 Results of ML Model

**FIGURE 2:**
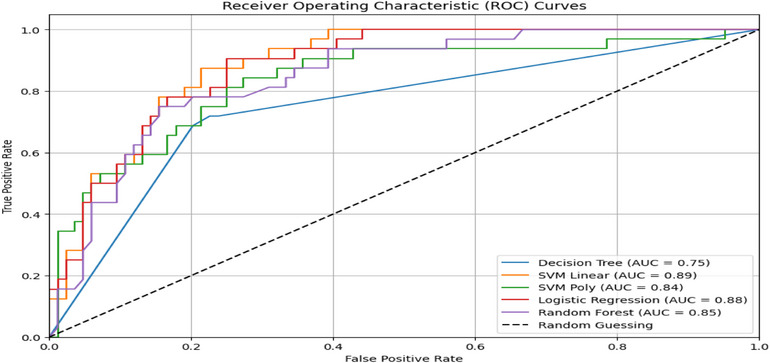
The ROC curves to predict internet addiction using DT, RF, SVM, and LR models. DT, decision tree; LR, logistic regression; RF, random forest; ROC, receiver operating characteristic; SVM, support vector machine.

The assessment of ML models, including DT, RF, SVM with the linear kernel (SVM‐linear), SVM with the polynomial kernel (SVM‐poly), and LR, encompassed the utilization of four key performance metrics from the confusion matrix (as detailed in Table 3), analysis of the area under the ROC curve (refer to Figure 2), and implementation of *k*‐fold cross‐validation techniques (as presented in Table 3). Assuming 70% observations as the training data and 30% observation as the test data with the set point 2023 runs using the scikit‐learn module, Table 3 showcases the calculated accuracy, sensitivity, specificity, and precision scores for DT, RF, SVM, and LR algorithms, providing valuable insights into their performance in classifying IA among adolescents.

**TABLE 3:** Accuracy, sensitivity, specificity, and precision of different machine learning (ML) models.

Indicators	DT	RF	SVM (polynomial kernel)	SVM (linear kernel)	LR
Accuracy	0.758	0.800	0.784	0.819	0.793
Sensitivity	0.718	0.750	0.687	0.687	0.593
Specificity	0.773	0.845	0.821	0.869	0.860
Precision	0.547	0.648	0.594	0.666	0.633

Abbreviations: LR, logistic regression; RF, random forest; SVM, support vector machine

Table 3 displays the performance metrics and their respective lower bound estimates for all models. For instance, the SVM linear model provided 81.9% of accurate classifications (i.e., accuracy = 0.819), 75% of positive cases that were classified as positive (i.e., sensitivity = 0.687), 86.9% of negative cases that were classified as negative (i.e., specificity = 0.869), and 66.6% of correct positive classifications (i.e., precision = 0.666).

### 4.4 Receiver Operating Characteristic Curve

Figure 2 displays the estimated AUC of DT, RF, SVM, and LR models, which were run using the scikit‐learn module in Python, considering 70% of observations as training data and 30% of observations as test data with random seed 2023. To classify the prevalence of IA within the last year among adolescents in Sylhet district, the estimated AUC was 0.75, 0.88, 0.84, 0.85, and 0.89 using ML models DT, LR, RF, SVM with a polynomial kernel of degree 2, and SVM with the linear kernel, respectively. The SVM linear kernel algorithm performed better with the maximum AUC among the examined ML algorithms.

### 4.5 *K*‐Fold Cross‐Validation Based on Accuracy

*K*‐fold cross‐validation was carried out for threefold, fivefold, and 10‐fold repetitions with random seed one and shuffle argument “True.” The findings are organized in Table 4. The SVM (linear kernel) model performed better in threefold, fivefold, and 10‐fold cross‐validations based on the higher accuracy scores, that is, 79.2%, 80.5%, and 80.4% to classify IA among adolescents within the last year, the SVM (linear kernel) algorithm performed better than DT, RF, SVM (polynomial kernel), and LR algorithms on the basis of accuracy measure, the ROC, and the *k*‐fold cross‐validation approaches.

**TABLE 4:** Result of accuracy based on *k*‐fold cross‐validation of machine learning (ML) models.

Models	Threefold	Fivefold	10‐fold
	MAss	MAss	MAss
DT	0.708	0.706	0.739
LR	0.791	0.801	0.799
SVM (polynomial kernel)	0.786	0.766	0.793
SVM (linear kernel)	0.792	0.805	0.804
RF	0.766	0.774	0.770

Abbreviations: LR, logistic regression; MAcc, mean of accuracy scores from each fold; RF, random forest; SVM, support vector machine.

### 4.6 Model Comparison

**FIGURE 3:**
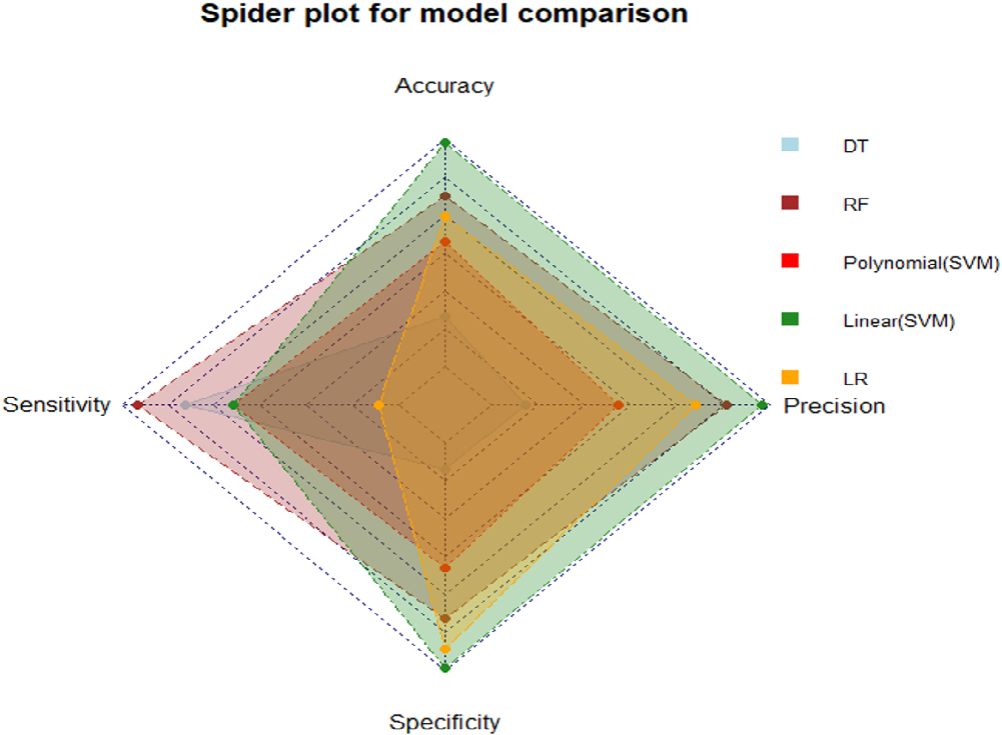
Spider plot of selected machine learning models across key performance metrics. DT, decision tree; LR, logistic regression; RF, random forest; ROC, receiver operating characteristic; SVM, support vector machine.

Figure 3 presents a spider plot comparing DT, RF, polynomial SVM, linear SVM, and LR models across accuracy, precision, sensitivity, and specificity. The SVM (linear kernel) model outperforms others with consistently high scores across all metrics, indicating strong overall performance. Linear SVM also shows competitive results, particularly in accuracy and precision. Polynomial SVM achieves high sensitivity but at the cost of slightly lower precision. DT shows moderate performance, whereas LR performs the weakest, especially in sensitivity.

### 4.7 Outcome Classification

Table 5 arranges this decision‐making for five students given data on their father's education, favorite activity, smoking status, internet games, internet use time, loneliness, and depression to classify their IA status using the fitted SVM (linear kernel) model.

**TABLE 5:** Prediction of internet addiction among adolescents using the fitted support vector machine (SVM) (linear kernel) model.

Features							Outcome prediction
Father's education	Favorite activity	Smoking status	Internet games	Internet use time (h)	Loneliness	Depression	Internet addiction status
College level	Others	No	Yes	3–4	Moderate	Yes	Addicted
School level	Gaming	No	Yes	3–4	High	Yes	Addicted
University level	Handing around	No	No	1–2	Low	No	Not addicted
College level	Use mobile phone	No	Yes	4–5	Moderate	Yes	Addicted
Don't know	Use mobile phone	Yes	Yes	4–5	High	Yes	Addicted

### 4.8 Factors Determined by LR Model

The fitted LR model illustrated that adolescent smokers are 3.9 (OR = 3.909, CI 1.008–15.16) times more likely to be addicted in comparison to nonsmokers. Adolescents who played internet games were 3.1 (OR = 3.164, CI 1.635–6.124) times more likely to be addicted in comparison to those who did not play internet games. Adolescents who used the internet for 2–3 h were 3.3 (OR = 3.362, CI 1.278–8.845) times more likely to be addicted in comparison to those who used the internet for less than 1 h. Adolescents who used the internet for 3–4 h were 6.3 (OR = 6.374, CI 2.382–17.055) times more likely to be addicted in comparison to those who used the internet for less than 1 h. Adolescents who used the internet for more than 6 h were 21 (OR = 21.453, CI 5.043–91.268) times more likely to be addicted in comparison to those who used the internet for less than 1 h. Adolescents who were depressed are 5 (OR = 5.013, CI 2.605–9.644) times more likely to become internet addicted in comparison to those who are not depressed, as shown in Table 6. In the LR model, other significant variables in the chi‐square analysis, such as internet use to connect close friends and loneliness, were insignificantly associated with IA.

**TABLE 6:** Odds ratios (OR) with 95% CI and *p* values obtained from the logistic regression model.

Variables	Category	OR	95% CI of OR		*p* value
			Lower	Upper	
**Father's education**	Don't know	Ref.	Ref.		Ref.
	Up to school level	0.553	0.224	1.366	0.199
	College level	2.137	0.832	5.486	0.114
	University	0.909	0.38	2.176	0.83
**Favorite activity**	Gaming	Ref.	Ref.		Ref.
	Hanging around	0.754	0.164	3.473	0.717
	Playing	0.807	0.168	3.883	0.79
	Sleeping	0.719	0.153	3.377	0.676
	Using mobile phone	4.627	0.793	26.998	0.089
	Others	0.333	0.059	1.867	0.211
**Smoking status**	No	Ref.	Ref.		Ref.
	Yes	3.909	1.008	15.16	0.049**
**Internet games**	No	Ref.	Ref.		Ref.
	Yes	3.164	1.635	6.124	0.001***
**Internet using time**	<1 h	Ref.	Ref.		Ref.
	1–2 h	1.838	0.733	4.607	0.194
	2–3 h	3.362	1.278	8.845	0.014***
	3–4 h	6.374	2.382	17.055	0***
	4–5 h	4.667	1.302	16.722	0.018***
	5–6 h	2.225	0.251	19.689	0.472
	>6 h	21.453	5.043	91.268	0***
**Internet use to connect with close friends**	No	Ref.	Ref.		Ref.
	Yes	2.133	0.453	10.04	0.338
**Loneliness**	Low	Ref.	Ref.		Ref.
	Modern	35,742,557	0		0.999
	High	87,006,232	0		0.999
**Depression**	No	Ref.	Ref.		Ref.
	Yes	5.013	2.605	9.644	0.00***

## 5 Discussion

Adolescents are particularly vulnerable to addiction and other mental health issues, and these challenges can have detrimental effects on both their overall well‐being and academic performance [39]. The worldwide prevalence estimates reveal varying degrees of addiction across different digital platforms: 26.99% for smartphone addiction, 17.42% for social media addiction, 14.22% for IA, 8.23% for cybersex addiction, and 6.04% for game addiction, as reported by Meng et al. [40]. Researchers found a significant proportion of IA among adolescents in India at 51%, Iran at 21.2%, Pakistan at 77.2%, and China at 58.8% [41, 42, 43, 44]. The prevalence of IA among Bangladeshi school‐going adolescents was 63% after COVID‐19. Another study reported that the overall prevalence of IA among students was 27.1% in Bangladesh [45, 46]. As a result, public concern for mental health issues among adolescents has been rising, and their IA has become a noticeable concept in psychological and mental health. Motivated by such a noticeable psychological and mental health concern, this research conducted a prevalence study to find the significant features and classification of IA among adolescents in Bangladesh using different ML models. This prevalence of our study showed that one‐third (30.1%) of adolescents reported IA within the last year. The study results showed that the adolescent father's education, favorite activity, loneliness, smoking status, depression, and internet use time were the most significant factors for their IA using the ML feature selection algorithm, Boruta. However, students’ fathers’ education, favorite activities, loneliness, smoking status, internet use to connect with close friends, depression, and internet use time were the significant factors for their IA using the conventional chi‐squared test.

The previous study reported that IA was significantly associated with living and physical activity, which are not matched with our current study, and smoking habit, a detached family relationship, time spent daily on the internet, and online gaming, which are matched with our current study (*p* < 0.05) among adolescents [45, 47]. When adolescents distance themselves from their families, it often disrupts communication among family members and leads to social issues, which can subsequently result in IA [48]. Heightened daily internet engagement among adolescents, notably on social media platforms such as Facebook and Twitter, precipitates addiction, consequently resulting in IA [49]. IA often experiences psychiatric symptoms, such as depression, and the literature supports the current study's findings [50, 51, 52, 53, 54, 55, 56]. A moderate positive association (*r* = 0.15 [95% CI: 0.13, 0.16]) was found between IA and loneliness, and the literature also supports our current study findings [57]. Prior studies indicate that the deterioration of intimate relationships correlates with declining mental health, leading to feelings of sadness and a defeated mindset. These can potentially trigger addictive behaviors linked to IA [48, 58]. Moreover, respondents with a smoking habit or minimal physical activity showed notably higher levels of IA [45].

The prior ML study reported that the classification accuracies were 66.67% for the IA status employing SVM in Indonesia and cent percent (mean absolute error and root mean square error are equal to zero) for classifying IA using JRip [59, 60]. Pertinent studies were undertaken to classify smartphone addiction, revealing that the RF model emerged with the highest accuracy among the various models employed [61, 62]. Other research focusing on mental health accuracy, 89.3% and 89% for stress and depression classification, outperformed employing the RF model, whereas SVM provided the best result than other models for the classification of anxiety (91.49% accuracy) in Bangladesh [63, 64]. However, our study found that the SVM (linear kernel) model performed better in all situations by using the highest mean estimates of performance parameters, that is, 89.3% accuracy, 86.9% specificity, 68.7% sensitivity, 66.6% precision, 89% AUC, and more than 80% accuracy in all threefold, fivefold, and 10‐fold cross‐validation techniques among adolescents because of different data sets. The SVM (linear kernel) model was used to classify IA among adolescents by considering the individual and interaction effects of all specified characteristics. Table 5 shows how any individual student's IA may be classified using the available data. On the other hand, the LR model failed to estimate the confidence interval for the one significant predictor variable, loneliness. This incomplete output is caused by incorrectly estimating the LR model. Before estimating the model, the LR model requires that all underlying assumptions be met, the most important of which are predictors having a strong correlation with the outcome variable and their independence (to prevent the multicollinearity problem). In this analysis, only a few variables, adolescents, will be used as a predictor variable in estimating the LR model correctly, as these variables were significantly associated (using the chi‐squared test in Table 1) with IA and had a significant association between them. Hence, to overcome the multicollinearity problem, only a few variables should be involved in estimating the LR model; otherwise, the results will be misleading. Furthermore, the SVM (linear kernel) model does not need any assumptions in estimating the model. As a result of its superior performance, the SVM (linear kernel) model will be more accurate and authentic (in terms of meeting the assumptions) in classifying IA among adolescents in the study.

### 5.1 Limitation and Strengthens

Furthermore, the major limitations of these analyses are the small sample size and the use of a random sample, which means that students in the survey may not represent the broader student population of Bangladesh. The study had another limitation due to its use of a cross‐sectional design, which might result in variations over time. Despite the study limitations, we feel our study has several appealing advantages or strengths in our research. The conventional chi‐square test identified seven variables as significant factors likely to result from the student's IA status. In contrast, the ML framework identified six variables as significant factors for classifying IA in this analysis more concisely. This work introduces the use of many ML models in the classification of IA, such as DT, SVM, and RF, which do not require any assumptions and are very simple (accessible) to use in any common program. Although the popular classifier LR demands that all underlying assumptions be met before estimating the model, predictors must be independent of each other and have a strong connection with the outcome variable. As a result, this commonly used prognostic modeling is difficult to estimate correctly, and incorrect estimates may result in some misleading data. The findings of our study can help researchers understand the limitations of the popular LR model for its assumptions’ constrained features. To execute the LR model authentically for this study, only a few factors must be included in classifying adolescent IA; otherwise, the classified model outputs will be correct but less informative.

### 5.2 Future Work

Future research should explore the incorporation of advanced ML algorithms like XGBoost, LightGBM, and CatBoost to enhance prediction accuracy by addressing both bias and variance. Expanding the study to longitudinal data would allow for the analysis of anxiety development over time, whereas integrating physiological and behavioral data could provide a more comprehensive understanding of IA. Additionally, applying the models to diverse student populations and developing real‐time monitoring tools could further broaden the impact. Future work could also focus on explainable AI (XAI) for improved transparency and explore the practical application of predictive models in policy‐making and educational interventions.

### 5.3 Recommendation

Our research findings indicated a notable incidence of IA among the adolescent participants. Consequently, it is important to enhance the level of knowledge among adolescents and their parents about the factors that might indicate the development of IA. With increased accuracy, the ML framework can be utilized to identify and classify significant prognostic indicators for this IA problem. This can aid policymakers, stakeholders, and families in comprehending and mitigating this critical crisis by enhancing policy‐making strategies and implementing effective counseling services.

## 6 Conclusion

The prevalence of excessive internet use among adolescents in Bangladesh is consistent with the worldwide trend. Several factors suggest a potentially concerning scenario, including depression, experiences of loneliness, and engagement in smoking behaviors, all functioning as strong markers of IA. Furthermore, there are noteworthy correlations between IA and factors such as participation in online gaming, duration of daily internet use, and level of physical activity. However, policymakers need to take proactive steps in addressing this matter by promoting early recognition and adopting efficient strategies to avoid and alleviate the possible hazards linked to excessive internet use. The ML framework can be utilized to identify noteworthy prognostic indicators, leading to more precise classifications for this IA problem. This, in turn, can aid policymakers, stakeholders, and families in comprehending and mitigating this critical crisis. Enhancing policy‐making strategies and implementing effective counseling services can facilitate the prevention of this crisis.

## Author Contributions

**Akher Ali**: conceptualization, formal analysis, data curation, writing – original draft, writing – review and editing. **Md. Sahadat Hosain**: data curation, validation, resources, writing – original draft, writing – review and editing. **Md. Abu Bakkar Siddik**: data curation, writing – original draft, writing – review and editing, supervision. **Md. Ahashan Habib**: formal analysis, writing – original draft. **Mahedi Hasan**: data curation, writing – original draft. **Al Mahmud**: data curation, writing – original draft. **Mohammad Mizanur Rahman**: writing – original draft. **Peal Ahamed Shanto**: writing – original draft. **Nafiul Hasan**: writing – original draft. **Mohammad Alamgir Kabir**: writing – review and editing, supervision.

## Funding

The authors have nothing to report.

## Conflicts of Interest

The authors declare no conflicts of interest.

## Data Availability

Due to the restrictions set by the ethical committee, the data will be provided upon reasonable request to the corresponding author.
